# Turkish Validity and Reliability of Short Form McGill Pain Questionnaire-2 in Patients with Chronic Cervical Radicular Pain Due to Disc Herniation

**DOI:** 10.5152/ArchRheumatol.2025.11133

**Published:** 2025-05-30

**Authors:** Ezgi Can, Rumeysa Çetinkaya Bulutoğlu, Hande Ece Öz, Furkan Çetin, Alp Eren Çelenlioğlu, Ender Sir

**Affiliations:** Clinic of Algology, Ankara Gülhane Research and Training Hospital, Ankara, Türkiye

**Keywords:** Chronic pain, neck pain, reliable, Short-Form McGill Pain Questionnaire-2, validity

## Abstract

**Background/Aims:**

:This study aimed to evaluate the reliability and validity of the Turkish edition of the Short-Form McGill Pain Questionnaire-2 (SF-MPQ-2) among chronic cervical radicular pain (CRRP) cases caused by disc herniation. The secondary aim of the study was to evaluate the relationship between Turkish Short-Form McGill Pain Questionnaire-2 (TR-SF-MPQ-2) and other pain and disability scales.

**Materials and Methods:**

:The study was based on data from 103 cases of CRRP patients evaluated at the Algology outpatient clinic. In addition to TR-SF-MPQ-2, the Numerical Rating Scale, Neck Disability Index, Quick Disabilities of the Arm, Shoulder, and Hand, Cervical Radiculopathy Impact Scale, and a 4-question neuropathic pain questionnaire were completed. Cronbach’s alpha (α) and intra-class correlation (ICC) tests were performed for reliability analyses. Confirmatory factor and Spearman correlation analysis were applied to assess structural and content validity, respectively.

**Results:**

Both the internal (α = 0.921) and test-retest reliability of the TR-SF-MPQ-2 were high (all ICC values >0.9 and *P* < .001) for the total and 4 subgroups (continuous, intermittent, neuropathic, and emotional). The total and subscale scores of the TR-SF-MPQ-2 were in correlation with other scale results (*r* = 0.404-0.648, *P* < .001). Confirmatory factor analysis demonstrated that the scale exhibited 4 distinct factors.

**Conclusion:**

The TR-SF-MPQ-2 is a valid and reliable scale for Turkish patients suffering from CRRP

**Main Points**

- The Short-Form McGill Pain Questionnaire-2 (SF-MPQ-2) is a multidimensional pain assessment tool that has been translated into many languages and has proven validity and reliability in many painful conditions.
- Chronic cervical radicular pain due to disc herniation is a common clinical condition in adult patients that significantly impairs the quality of life.
- Our study demonstrated that the Turkish version of the SF-MPQ-2 is a valid and reliable questionnaire for the multidimensional assessment of chronic cervical radicular pain, patient management, and follow-up.

## Introduction

Chronic neck pain is a clinical condition that persistsfor a minimum 3 months and significantly worsens quality of life, occurring in nearly one-third of adults.^1^ Radicular pain is characterized by an electrical, burning, and discomfort that radiate from the neck to the upper limbs. This is caused by compression and irritation of the nerve root and dorsal root ganglion due to a cervical hernia or osteophytes in the cervical spine and presents both nociceptive and neuropathic characteristics.^1,2^ Approximately 30%-40% of patients with chronic pain radiating from the neck to the upper extremities have neuropathic features.^3-5^ The coexistence of different pain types, such as neuropathic and nociceptive pain, and psychosocial factors leads to chronic pain, which affects the treatment and prognosis of cervical radicular pain.^5^

Unidimensional measurement methods, including the visual analogue, vocal, and numeric rating scales, are widely used to determine pain severity. However, these measurement methods do not provide sufficient data on the emotional and psychosocial components of pain other than intensity.^6^ The difficulty and complexity of assessing pain sensation has resulted in the development of multidimensional pain scales. The McGill Pain Questionnaire (MPQ) is a multidimensional scale that is frequently utilized to describe complex characteristics of chronic pain.^7^ Owing to the length of the MPQ and its difficulty in daily practice, the Short Form McGill Pain Questionnaire-2 (SF-MPQ-2) developed by Dworkin and colleagues, to which neuropathic pain characteristics were added, has become widely used.^8^

The SF-MPQ-2assesses patient’s pain sensation by dividing it into 4 subgroups in terms of its persistence, intermittency, neuropathic, and emotional characteristics.^8,9^ It is a comprehensive questionnaire that has been translated into many languages and has been proven to be valuable and useful in many pain conditions, asking about the type and intensity of pain, as well as neuropathic and emotional domains (musculoskeletal pain, visceral pain, cancer pain, neuropathic pain, rheumatoid arthritis, osteoarthritis).^6,10-17^

The SF-MPQ-2 was translated into Turkish and this version of the SF-MPQ-2 (TR-SF-MPQ-2) has been shown to be valid and reliable in patients with chronic low back pain.^17^ The principal objective was to evaluate the validity and reliability of the Turkish version of this scale in chronic cervical radicular pain (CRRP) due to herniated disc. The secondary objective was to determine the association between the TR-SF-MPQ-2 and the other measurement scales.

## Materials and Methods

### Study Design and Participants

The present prospective validity and reliability study was evaluated and accepted by the Gülhane Research and Training Hospital medical ethics board (no. 2024/81; date: 14.11.2024) and was completed in line with the principles of Helsinki. Scales were administered to patients with CRRP who presented to the Gülhane Research and Training Hospital Algology outpatient clinic between November 2024 and January 2025. Permission was obtained from Dworkin RH and Mapi Research Trust prior to using the TR-SF-MPQ-2 questionnaire to assess its validity and reliability.^8^

After informed consent was received, 127 patients were enrolled in the analysis, after which data from 7 patients with cervical canal stenosis, 8 with diabetes mellitus, and 9 with carpal tunnel syndrome were excluded. The study included 103 patients with CRRP due to herniated disc. According to the COSMIN guideline, a sample size of ≥100 for quantitative studies (questionnaires) is considered “very good” and 103 patients were included in our study.

**Figure 1.**
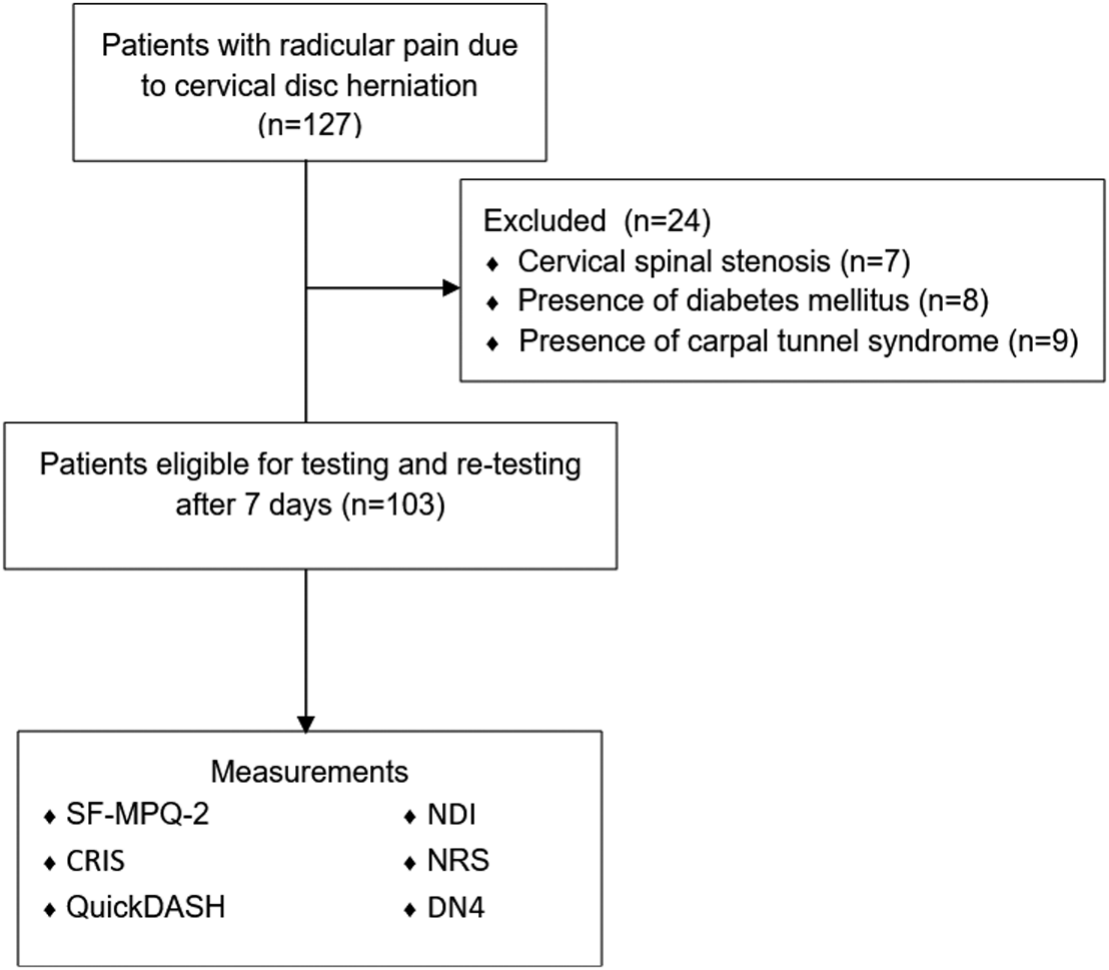
Study design and flowchart.

When the inclusion criteria are detailed: cervical radicular pain for longer than 3 months, being the age of 18 years or more, being literate in Turkish, being cooperative in responding to the scales, and the presence of disc herniation causing cervical radicular pain shown by magnetic resonance imaging (MRI) and patients with normal neurological (motor, sensory, and reflex tests) and physical examination. The exclusion criteria included a range of medical conditions, including cervical canal stenosis as demonstrated by MRI, neuromuscular and rheumatological diseases, diabetes mellitus, upper limb entrapment neuropathy, abnormal motor and sensory examination findings, illiteracy, and neurological or mental diseases affecting coordination and orientation. A flow diagram illustrating the study methodology is presented in Figure 1.

Patients’ age (years), gender, period of illness (months), location of pain (unilateral/bilateral), medications used, previous treatments, and level of cervical disc herniation were documented. The TR-SF-MPQ-2 is a scale of which a Turkish version is available and a validation study was completed in patients with chronic low back pain.^17^ In this study, this scale already translated into Turkish was used (supplementary material-1). For the validation of this scale in CRRP patients, Numerical Rating Scale (NRS), Quick Disabilities of the Arm, Shoulder, and Hand (QuickDASH), Neck Disability Index (NDI), 4-question neuropathic pain questionnaire (DN4), and cervical radiculopathy impact scale (CRIS) were administered to all patients at their first visit. The NDI and QuickDASH were used to assess patients’ daily living and disability levels,^18,19^ while the CRIS was utilized to estimate the impact of the cervical pain radiating to the arm on functioning.^20^ The NRS was used to determine pain severity.^18^ The DN4 assessed the neuropathic etiology of complaints and findings.^19^ In addition, the TR-SF-MPQ-2 was re-completed and retested after 7 days (face-to-face).

### Scales

#### Short-Form McGill Pain Questionnaire-2:

The SF-MPQ-2 is comprised of 4 separate categories of pain (affective, continuous, intermittent, and neuropathic pain) and a total of 22 pain descriptors. Affective pain descriptors include “tiring, exhausting,” “nauseating,” “frightening,” and “punishing-cruel”; continuous pain descriptors include “throbbing,” “cramping,” “gnawing,” “aching,” “severe pain,” and “tender pain”; intermittent pain descriptors include “shooting,” “stabbing,” “electric shock,” “sharp,” “piercing,” and “splitting pain”; and neuropathic pain descriptors include “hot burning,” “cold freezing,” “pain with light touch,” “itching,” “tingling or pricking,” and “numbness.” The severity of these pain characteristics was scored on a 0-10 scale. In the questionnaire, scores were calculated for subcategories and in total.^8,17^ The TR-SF-MPQ-2 is a scale of which a Turkish version is available and a validation study was completed in patients with chronic low back pain.^17^ In this study, this scale already translated into Turkish was used (supplementary material-1).

#### Numerical Rating Scale:

The verbal NRS is a commonly used, easy-to-understand scale that the patient scores between 0 and 10 according to the severity of pain.^21^

#### Quick Disabilities of the Arm, Shoulder, and Hand:

This questionnaire measures patients’ functionality and symptom perception associated with pathology affecting the upper limb and consists of 11 components. The scale, which is scored between 0 and 100, has been tested in Turkish patients.^19^

#### Neck Disability Index:

This scale estimates the effect of neck pain on activity during the day and performance and has been validated in Turkish patients.^22^ It is a 10-component measuring tool, with a high score indicating an increased degree of disability.

#### Cervical Radiculopathy Impact Scale:

The questionnaire measures the impact of radicular pain with arm and neck symptoms (pain, numbness, and loss of sensation) on functioning. It is scored from 0 to 100, with an increased score indicating decreased functioning.^20,23^

#### 4-Question Neuropathic Pain Questionnaire:

It is a test used to determine the presence of neuropathic pain. It assesses 7 items associated with neuropathic characteristics of pain and 3 components related to the examination. The total score ranges from 0 to 10 to >4 points, indicating the presence of neuropathic pain.^24^

### Statistical Analysis

The analyses were executed utilizing IBM SPSS Statistics for Windows, Version 23.0 (IBM SPSS Corp.; Armonk, NY, USA). Histogram, normality plots, and Kolmogorov–Smirnov normality tests were performed to assess the distribution of the data. According to the findings of this analysis, the dataset does not conform to normal distribution. Descriptive data were presented as median, interquartile range, minimum, maximum, frequency, and percentage. A value of *P* < .05 indicates that the result of the analysis is statistically significant.

### Reliability Analysis

The reliability of the scale was analyzed in 2 parts. The first was an internal consistency test utilizing Cronbach’s alpha (α) coefficient, while the second was a test-retest reliability test employing the intra-class correlation coefficient (ICC) and a 95% CI. A Cronbach’s *α* coefficient >0.7 was regarded as acceptable. The ICC is evaluated as weak reliability with less than 0.5, intermediate reliability between 0.5 and 0.75, good reliability between 0.75 and 0.9, and perfect reliability with more than 0.90.^25,26^

### Validity Analysis

The validation of TR-SF-MPQ-2 was evaluated utilizing confirmatory factor analysis (CFA) and Spearman’s correlation analysis. For CFA, chi-square/degree of freedom (C^2^/*df*), goodness of fit index (GFI), comparative fit index (CFI), normed fit index (NFI), and root mean square error of approximation (RMSEA) were calculated. If the C^2^/*df* ratio was less than 3, the model showed a very strong fit. The GFI, CFI, and NFI values greater than 0.90 are considered adequate and RMSEA less than 0.10 is an evidence of a valid fit.^8^ Correlation coefficients (rho) between the 4 subcategory scores of TR-SF-MPQ-2 and NRS, CRIS, QuickDASH, NDI, and DN4 scores were determined. The coefficients were evaluated as very good (0.91-1.00), good (0.71-0.90), intermediate (0.51-0.70), adequate (0.31-0.50), and poor (<0.30).^26^

## Results

This study analyzed data from 103 cases of CRRP due to herniated disc. The numeric and descriptive characteristics of the participants are presented in Table 1. Pain characteristics were analyzed based on TR-SF-MPQ-2 subgroup scores and 62.1% persistent, 66.1% intermittent, 57.2% neuropathic, and 66.1% affective pain was found. The scores for the baseline TR-SF-MPQ-2 (total), NRS, QuickDASH, NDI, CRIS, and DN4 are shown in Table 2.

### Reliability

The reliability of the scale was analyzed according to Cronbach’s alpha (α); high internal consistency values were obtained for total scores and subscales (*α* = 0.921). Secondly, inter-class reliability was assessed and found to be high for all scores (*P* < .001) (Table 3).

### Validity

The overall and subgroup scoring data of the TR-SF-MPQ-2 demonstrated a correlation with the NRS, QuickDASH, NDI, CRIS, and DN4 scores, providing support for the content validity of the scale (*r* = 0.404-648, *P* < .001), as shown in Table 4.

To evaluate the internal construct validity of the scale, CFA was conducted. Following the rotation of the factor loadings using the direct oblimin rotation method, 4 subscales were identified (Table 5). The results of CFA, chi-square/degree of freedom (C^2^/*df*), GFI, CFI, NFI, and RMSEA indicated a valid fit (Table 6).

### Discussion

Chronic cervical radicular pain is a clinical condition that is common in adults and frequently leads to disability with nociceptive, neuropathic, and emotional components.^1,3,5^ The chronic and multi-dimensional nature of the disease necessitates a detailed evaluation in the initial assessment and follow-up of the patients. Therefore, scales such as the TR-SF-MPQ-2, which evaluates pain in a multidimensional way and considers psychological aspects in addition to pain, play an important role in disease management and follow-up in patients with chronic cervical pain.

The validity and reliability of TR-SF-MPQ-2 for CRRP patients were evaluated by applying internal consistency, test-retest, and factor evaluations. Cronbach’s *α* values were >0.9 for overall and 4 subscales, indicating a high internal consistency and consistent with the initial English version and other translated versions.^8,11,12,27,28^ The intra-class correlation values calculated using the test-retest method were 0.937, 0.988, 0.933, and 0.969 for the continuous, intermittent, neuropathic, and affective subscales, respectively, demonstrating excellent reliability. These correlation values are similar to those reported for the initial SF-MPQ-2 and other translated versions.^8,17,27^ To evaluate the construct validity and to determine the possible correlations of the items, CFA was applied with the same 4 subscales used in similar studies and in the initial English version. A 4-factor model was obtained that fit well with the findings from patient answers about CRRP.^8,11,27-29^

The SF-MPQ-2 is a multi-faceted pain questionnaire frequently utilized worldwide for many acute and chronic painful conditions and has been adapted to many languages, and reliability and validity researches have been conducted.^8-14^ Aykan et al^17^ demonstrated its validity and reliability in Turkish patients with chronic low back pain. The present research assessed the suitability of the questionnaire for use in patients with CRRP, a chronic painful condition that is often difficult to manage. The questionnaire includes sensory and emotional characteristics and addresses both the persistent and intermittent nature of pain.

Numerical Rating Scale is a pain scale that measures the severity of pain and has been shown to be valid and reliable.^21^ There was a moderate correlation (*r* = .553) between the NRS and the total and subgroup scores of the TR-SF-MPQ-2 in the concurrent validity analysis, supporting the results of previous studies.^6,17^ In addition, SF-MPQ-2 subscale scores were acceptable and showed also positive and significant correlation with NDI (*r* = 0.546), QuickDASH (*r* = 0.648), and CRIS (*r* = 0.595) scale scores, which provides insight into disability and functionality due to CRRP. This correlation was similar, moderate, and significant in all 4 subscale groups of the scale (the values range from 0.404 to 0.648). Similarly, studies conducted in other chronic pain conditions have also shown significant correlations between other disability and functionality scale scores.^12,16,17,28^ These findings recommend that the TR-SF-MPQ-2 scale can be safely used to predict the severity of CRRP and the effect of pain on functioning in Turkish patients.

In this study, a positive and moderate correlation (*r* = 0.594) was found between the subscale of the TR-SF-MPQ-2, which evaluates the neuropathic features of pain, and DN4 scores.^2^ Studies have shown that the neuropathic subscale of this questionnaire has a positive correlation with the results of neuropathic pain scales such as the Identification pain questionnaire (ID pain), as well as DN4.^12,17,29,30^ Therefore, the neuropathic subgroup scores of TR-SF-MPQ-2 may be considered a marker for the presence of neuropathic pain, which is frequently accompanied by CRRP (30%-40%) and is important for pain management.^5^

The present study had several limitations. First, it only included patients suffering from CRRP from a single center. Second, the data from patients in the acute and subacute phases were not included. Third, if any treatment was applied to the patients, the effect of the scale scores on post-treatment changes could not be evaluated. Conducting multicenter studies incorporating treatment and follow-up designs would enhance the assessment of the questionnaire’s benefits; however, the study’s strength lies in the homogeneity of the patient cohort. However, to the best of knowledge, this is the first research conducted to investigate the validation and reliability of the TR-SF-MPQ-2 in the Turkish population with CRRP secondary to disc herniation.

In conclusion, the results of this study showed how reliable, valid, and sensitive the TR-SF MPQ-2 is as a tool for the assessment of CRRP. The questionnaire is able to distinguish various types of pain, including affective and neuropathic features, and may assist clinicians in the management and follow-up of patients by offering a multidimensional approach to pain assessment. This scale can be regarded as a valuable instrument in pain clinics for the evaluation of patients with CRRP, in terms of pain characteristics, appropriate therapeutic selection, and response monitoring.

## Figures and Tables

**Table 1.** Demographic and Clinical Features of the Patients

Variables (n = 103)	Results
Age (median, min-max/mean ± SD)	51 (28-81)/51.5 ± 11.9
Sex (female/male)	64 (62.1%)/39 (37.9%)
Pain duration, months (median, min-max / mean ± SD)	24 (3-360)/46.1 ± 65.6
**Pain Side, n (%)**	
Right	27 (26.2%)
Left	36 (25.2%)
Bilateral	50 (48.5%)
Analgesic use, n (%)	
NSAID	25 (24.3%)
Opioid	5 (4.9%)
Anticonvulsant	4 (3.9%)
Antidepressants	5 (4.9%)
Multiple drugs	45 (43.7%)
Non drug	19 (18.4%)
Previous treatments, n (%)	
Oral analgesic drug	27 (26.2%)
Physiotherapy	8 (7.8%)
Interventional pain treatments	2 (1.9%)
Multiple treatments	61 (59.2%)
None treatment	5 (4.9%)
Affected nerve root level, n (%)	
One level	50 (48.5%)
Multiple levels	53 (51.5%)

Data are expressed as median values (min-max) or number of patients (%).

NSAID, non-steroidal anti-inflammatory drug; SD, standard deviation.

**Table 2.** Scale scores of the patients (n=103)

Scale	Mean ± SD Median, min-max
NRS	8.1 ± 1.2 8 (5-10)
QuickDASH	59.2 ± 21.2 59.0 (11.2-100.0)
NDI	26.1 ± 10.2 25 (3-50)
CRIS	70.2 ± 15.9 69 (32-98)	
DN	44.5 ± 1.9 5 (0-9)	
TR-SF-MPQ-2, Total	5.5 ± 3.5 6 (0-10)

CRIS, cervical radiculopathy impact scale; DN4, 4-question neuropathic pain questionnaire; NDI, neck disability index; NRS, numerical rating scale; QuickDASH, Quick Disabilities of the Arm, Shoulder, and Hand; SD, standard deviation; TR-SF-MPQ-2, short form McGill Pain Questionaire-2.

**Table 3.** Internal Consistency and Test-Retest Reliability of TR-SF-MPQ-2 Scale (Subscales and Total)

	T1	T2-Retest	Cronbach’s *α*	ICC (95% Cl)	*P*
	(Mean ± SD)	(Mean ± SD)	Coefficient		
Continuous	5.4 ± 3.5	5.4 ± 3.4	0.862	0.937 (0.917-0.953)	**<.001**
Intermittent	5.9 ± 3.2	5.8 ± 3.2	0.973	0.988 (0.984-0.991)	**<.001**
Neuropathic	4.8 ± 3.7	4.8 ± 3.6	0.851	0.933 (0.912-0.951)	**<.001**
Affective	5.9 ± 3.6	6.0 ± 3.5	0.928	0.969 (0.959-0.977)	**<.001**
Total	5.5 ± 3.5	5.5 ± 3.4	0.921	0.981 (0.976-0.986)	**<.001**

ICC, intra-class correlation coefficients; SD, standard deviation; T1, test-1; T2, Re-test. Bold p-values indicate statistical significance.

**Table 4.** Correlations Between TR-SF-MPQ-2 and Other Scale Scores and Content Validity Results

	Continuous	Intermittent	Neuropathic	Affective	Total
	*r* *P*	*r* *P*	*r* *P*	*r* *P*	*r* *P*
NRS	0.554 <.001	0.516 <.001	0.527 <.001	0.553 <.001	0.553 <.001
QuickDASH	0.597 <.001	0.577 <.001	0.648 <.001	0.599 <.001	0.648 <.001
NDI	0.546 <.001	0.469 <.001	0.538 <.001	0.501 <.001	0.546 <.001
CRIS	0.595 <.001	0.546 <.001	0.641 <.001	0.555 <.001	0.595 <.001
DN4	0.439 <.001	0.447 <.001	0.594 <.001	0.404 .001	0.564 <.001

CRIS, cervical radiculopathy impact scale; DN4, 4-question neuropathic pain questionnaire; NDI, neck disability index; NRS, numerical rating scale; QuickDASH, Quick Disabilities of the Arm, Shoulder, and Hand; r, Rho.

**Table 5.** Results of Standardized Factor Loadings According to Confirmatory Factor Analysis

	Continuous	Intermittent	Neuropathic	Affective
1. Throbbing pain	0.515			
2. Shooting pain		0.913		
3. Stabbing pain		0.971		
4. Sharp pain		0.930		
5. Cramping pain	0.715			
6. Gnawing pain	0.392			
7. Hot-burning pain			0.530	
8. Aching pain	0.333			
9. Heavy pain	0.528			
10. Tender	0.529			
11. Splitting pain		0.965		
12. Tiring-exhausting				0.666
13. Sickening				0.615
14. Fearful				0.746
15. Punishing-cruel				0.698
16. Electric-shock pain		0.810		
17. Cold-freezing pain			0.789	
18. Piercing		0.846		
19. Pain caused by light touch			0.436	
20. Itching			0.696	
21. Tingling or “pins and needles”			0.896	
22. Numbness			0.847	

Principal component analysis, rotation method: Direct oblimin, Kaiser–Meyer–Olkin Measure of Sampling Adequacy: 0.914 and *P* < .001.

**Table 6.** Fit Index Values for Confirmatory Factor Analysis of Subgroup Scores of TR-SF-MPQ-2

	*X^2^/df*	GFI	CFI	NFI	RMSEA
Continuous	2.546	0.948	0.962	0.927	0.046
Intermittent	2.345	0.932	0.953	0.918	0.058
Neuropathic	1.923	0.974	0.981	0.933	0.032
Affective	2.013	0.965	0.956	0.924	0.045

CFI, comparative fit index; *df*, degree of freedom; GFI, goodness fit index; NFI, normative fit index; RMSEA, root mean square error of approximation; TR-SF-MPQ-2, Turkish Short form McGill Pain Questionnaire; *χ^2^*, Chi-square.

## Data Availability

The data that support the findings of this study are available on request from the corresponding author.
